# Using Momentary Measures to Understand Physical Activity Adoption and Maintenance: Protocol for a Longitudinal Study

**DOI:** 10.2196/108686

**Published:** 2026-09-25

**Authors:** Neng Wan, Wonwoo Byun, Ming Wen, Emre Ertin, Jeff Phillips, Nia Aitaoto, Simon Brewer, David W Wetter

**Affiliations:** 1 School of Environment, Society and Sustainability, University of Utah, 260 S Central Campus Dr, Salt Lake City, UT, 84112, United States, 1 8015853972; 2 Department of Health & Kinesiology, University of Utah, Salt Lake City, Utah, United States; 3 Department of Sociology, The University of Hong Kong, Hong Kong, China (Hong Kong); 4 Department of Electrical and Computer Engineering, The Ohio State University, Columbus, OH, United States; 5 School of Computing, University of Utah, Salt Lake City, Utah, United States; 6 Association of Asian Pacific Community Health Organizations, Washington, DC, United States; 7 Department of Population Health Sciences, University of Utah, Salt Lake City, Utah, United States

**Keywords:** mobile health, mHealth, physical activity, PA, PA adoption, PA maintenance, ecological momentary assessment, EMA, geographic information system

## Abstract

**Background:**

Physical inactivity is prevalent among adults in the United States and is related to various health disparities. The search for effective policies and interventions to promote physical activity (PA) is severely hampered by the paucity of research on the mechanisms underlying PA behavior change.

**Objective:**

This paper describes a research protocol that uses mobile health technology to examine the influence of contextual and environmental factors and acute momentary precipitants on PA adoption and maintenance among Pacific Islanders in the United States.

**Methods:**

The study is guided by an overarching conceptual framework derived from models of the social and environmental determinants of health, social cognitive theories of behavior change, and prior empirical findings. Participants will be assessed using real-time, field-based, state-of-the-art methodologies consisting of MotionSense, ecological momentary assessment, and GPS tracking. MotionSense tracks behavioral and physiological data in real time and can objectively detect PA behaviors of participants. GPS tracking permits real-time mapping of an individual’s space-time trajectories and relevant environmental exposures and characteristics (eg, proximity to PA facilities and neighborhood safety) using Geographic Information System data. Principal outcomes of interest are PA adoption and PA maintenance.

**Results:**

This study was funded by the National Cancer Institute of the National Institutes of Health in August 2023. Data collection started on May 23, 2025, and is expected to finish by March 2028. As of August 10, 2026, the project has recruited all 150 participants. Data analysis is ongoing, and results are expected to be published in May 2028.

**Conclusions:**

This is among the first studies to link objective and momentary indexes of PA to key environmental and psychosocial factors in PA behavior studies. The comprehensive, multi-method approach addresses 2 long-standing limitations in PA research: the reliance on self-reported outcome measures and the use of static residential locations as proxies for neighborhood exposure. In addition, this study is among the first to apply dynamic prediction models, a novel statistical approach well suited to the high-frequency, intensive longitudinal data generated by real-time mobile health assessment. The findings will provide actionable evidence to inform policies and interventions aimed at reducing PA-related health disparities among Pacific Islanders and other racial and ethnic groups that experience similar health problems.

## Introduction

### Background

Physical activity (PA) is important for reducing obesity [1-3] and the risk of chronic conditions such as cancer, diabetes, and cardiovascular disease [2,4,5]. However, physical inactivity remains a persistent public health challenge in the United States, with over 25% of adults reporting no PA outside of work [6] and some racial and ethnic minority groups and low–socioeconomic status populations being even less physically active than other groups [7]. Policies and interventions that help these population groups increase and maintain their PA level would help reduce the enormous racial, ethnic, and socioeconomic disparities in obesity and obesity-related health conditions.

A critical step on PA promotion is to understand the mechanisms underlying PA adoption and maintenance. A consistent challenge for PA interventions is how to maintain participants’ PA levels after the program is over, as ongoing participation in the behavior is necessary to achieve long-term health benefits [8,9]. Most PA studies show that programs are successful in getting people to start exercising but maintenance is poor. Typically, only half or less of those who start an intervention maintain their gained PA level after 6 months [8,10].

Various theories and models, including social cognitive theory [11] and social ecological models [12,13], demonstrate the importance of social and built environments in shaping PA behaviors and the role of psychosocial factors (eg, self-efficacy and affect) as mediators [14-17]. Built and social environment features can facilitate or hinder PA by shaping cognitive mediators such as self-efficacy and motivation [18]. A thorough understanding of such mechanisms should be based on a microtemporal scale (eg, hourly, within-day, or cross-day data that can capture the dynamic interactions among contextual factors, psychosocial factors, and PA behaviors) [19]. However, such data have seldom been collected for any population in previous studies. The lack of real-time, real-world measures of PA and PA determinants has precluded the development of evidence-based interventions and policies to reduce the disproportionate health burden of high-risk population groups [15,20-22].

### Objectives and Aims

This paper describes the protocol of a research project that uses mobile health (mHealth) technologies to understand momentary mechanisms underlying PA behaviors as well as longer-term PA adoption and maintenance. The longitudinal study, named project PiPA (Pacific Islander Physical Activity), focuses on PA behaviors among Pacific Islanders in the United States, a high-obesity but physically inactive population group. Specifically, it uses smartphone GPS tracking, wrist-worn accelerometers, geographic information systems (GIS), and ecological momentary assessment (EMA) to examine the influence of neighborhood factors and momentary psychosocial and contextual factors on PA behavior change among 150 previously sedentary Pacific Islander adults in Utah hoping to identify contextual and psychosocial mechanisms to better inform future PA interventions for Pacific Islanders and other sedentary groups. The aims of the PiPA study are to (1) examine the influences of neighborhood factors on PA adoption and maintenance among sedentary Pacific Islander adults living in Utah and (2) determine the dynamic relationships between contextual and psychosocial acute precipitants and PA among sedentary Pacific Islander adults living in Utah.

## Methods

### Study Design and Setting

**Figure 1.**
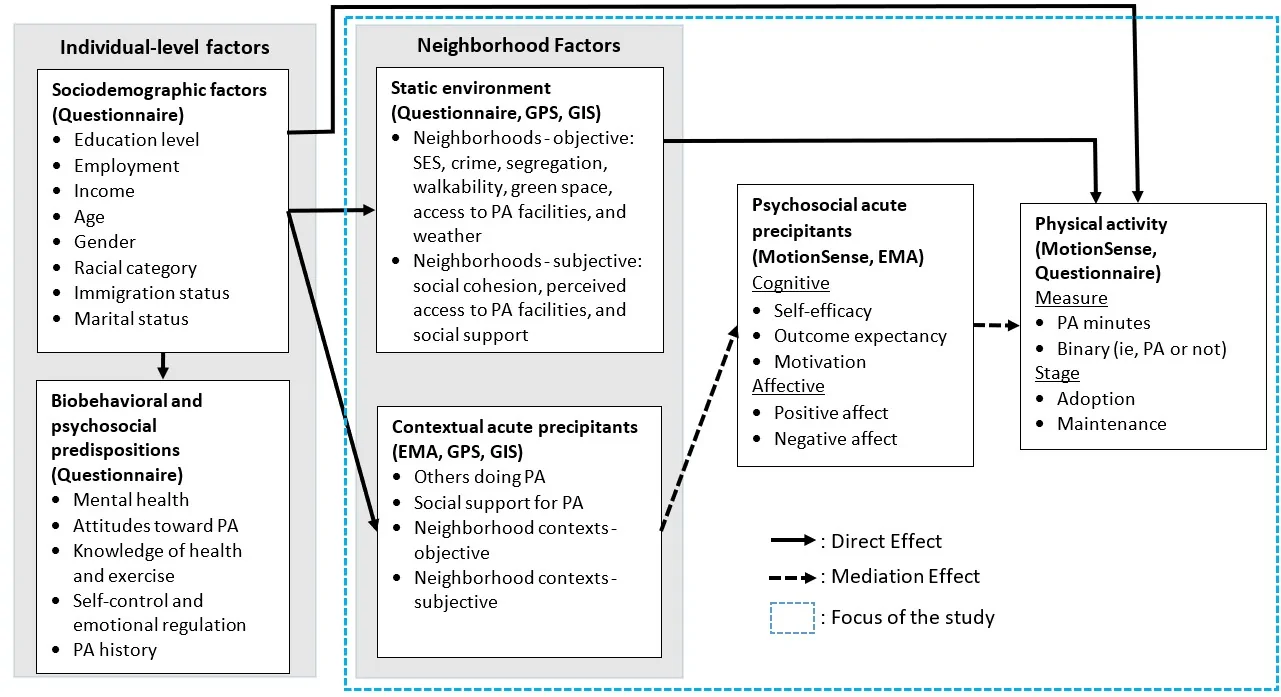
Conceptual framework of the study. EMA: ecological momentary assessment; GIS: geographic information system; PA: physical activity; SES: socioeconomic status.

This longitudinal study includes an evidence-based PA program provided to all participants to help improve PA levels and then study how contextual and psychosocial factors influence PA initiation and maintenance. Participants will be assessed for 5 separate weeks during a 21-month span using real-time, field-based, state-of-the-art methodologies consisting of MotionSense (a well-validated on-body mobile sensor system) [23], EMA, and smartphone GPS—all collected through a mobile and cloud software stack named MotionPI [24]. These units collect real-time momentary data in natural environments, communicate wirelessly with each other, and process data in real time on the smartphone. MotionSense continuously measures an individual’s PA behavior, negative affect, and stress based on specific physiological “signatures” without requiring any volitional action by the individual. Using real-time communication between these systems, EMAs, which assess subjective experience, can be triggered through detection of PA, as well as via random assessments. GPS tracking, which runs in the background on the smartphone, permits real-time spatial mapping of location patterns and determining key PA characteristics (eg, trip purpose and PA type). MotionSense and EMA data will be paired with GPS coordinates, which can then be associated with spatially and temporally relevant characteristics of the built (eg, access to PA facilities and public transit, street walkability, and green space) and social (eg, poverty and residential segregation) context using GIS data. The ability to objectively and continuously capture PA and its key correlates while requiring minimal action from participants could provide more accurate and finer temporal-scale information regarding PA behaviors than previous studies. Principal outcomes of interest are PA adoption and maintenance over extended periods, as well as momentary PA indicators. Thus, key pathways can be generated that link contextual social and built environmental characteristics to PA behaviors through psychosocial factors (Figure 1).

### Conceptual Framework

Key theoretical constructs, pathways, assessment methodologies, and analytic strategies of this proposed study are guided by an overarching conceptual framework derived from models of the social and environmental determinants of health, social cognitive theories of PA behavior change [25], and prior empirical findings [25-27]. Figure 1 shows a modeling framework that guides our analysis. Contextual built and social factors and social cognitive and affective acute precipitants, along with individual sociodemographic factors and biobehavioral and psychosocial predispositions, are modeled as contributors to PA. The focus of the proposed project is to understand how neighborhood factors are associated with PA behavior change (ie, adoption and maintenance) among previously sedentary Pacific Islanders (aim 1) and how momentary contextual and psychosocial factors influence PA behaviors (aim 2). It is necessary to distinguish PA adoption from PA maintenance because studies in the general population show that PA correlates and determinants may differ between the 2 stages.

### Study Population

Our study population, Pacific Islanders in the United States, experiences disproportionately high rates of obesity [28,29] and associated chronic diseases, including cardiovascular diseases [30], diabetes [31], and various types of cancer [32], compared with the general US population. In addition, Pacific Islanders consistently report lower PA engagement and are less likely to meet national PA guidelines compared to non-Hispanic White individuals [33]. Unfortunately, Pacific Islanders are frequently included within a larger Asian American grouping in public health research. Asian Americans as a whole generally have more favorable health profiles [34]. As such, this practice obscures Pacific Islanders’ distinct health risks and lived experiences [14,35].

Given that there are multiple Pacific Islander groups with different areas of origin and cultural backgrounds, this study focuses on only 1: Tongan American adults in Utah. Utah has the highest per capita percentage of Pacific Islanders among the continental states, with a Pacific Islander population of 38,000 in 2014 [36]. Tongan Americans are the largest Pacific Islander group in Utah, accounting for 38% of the total Pacific Islander population [37-39]. It is hoped that this study will provide a template to study other Pacific Islander groups in the future.

### Participants and Recruitment

This longitudinal cohort study will enroll 150 sedentary Tongan American adults (equally split between male and female individuals) in Utah. Tongan Americans primarily live in urban areas of the Salt Lake and Utah counties, the 2 most populous counties in the state. Approximately 7% of Tongan Americans hold a bachelor’s degree [39], the lowest rate among all racial and ethnic groups. Although most live in relatively low-income neighborhoods, over a quarter live in census tracts with poverty rates of less than 5% (average median household income US $92,301) [39], suggesting good heterogeneity in their socioeconomic status. Our preliminary analyses also show good variations in crime rate, park density, green space, and land use mix among these census tracts (N. Wan, unpublished data, September 2021). Approximately 27% of the Tongan American population in Utah is foreign born [39]. Although few studies have examined PA behaviors of Tongan Americans specifically, our previous focus group study on 36 Tongan American adults found that most of them were physically inactive (ie, 86% of them had <120 minutes of moderate to vigorous PA [MVPA] per week, and 33% had <30 minutes of MVPA per week).

Participants will be recruited through a community-engaged, multipronged strategy that combines media outreach (eg, via public service announcements, radio and TV interviews, feature stories in print media, and Tongan American–oriented radio and television) and community-based outreach supported by our long-standing research partnerships within the Tongan American community. We will recruit approximately 15 eligible participants per month over 10 months.

Participants will attend a baseline visit at the Center for HOPE at the University of Utah to complete eligibility screening. Our inclusion criteria are 18 years of age or above; being Tongan American; being currently sedentary (defined as self-reported PA of less than 3 days per week for less than 20 minutes per day over the previous 6 months [25]); valid home address and telephone number; ability to speak, read, and write in English; and marginal or adequate health literacy. Exclusion criteria are a pacemaker or implanted cardiac device and any dietary or exercise restrictions and/or health conditions that would preclude them from fully participating. Eligible individuals who pass screening will provide informed consent and then complete a baseline questionnaire, undergo anthropometric measurements, and receive training on the study devices. Recruitment will be conducted in close collaboration with the National Tongan American Society throughout the study. Four Tongan American community members will serve on a community advisory board to support outreach and retention efforts.

### Sample Size and Power

The targeted enrollment (N=150) was selected to provide adequate power for studying both momentary and long-term outcomes (PA adoption and maintenance). Power simulations indicate that, with 25% to 30% attrition, the study remains sufficiently powered (≥80%) to detect small to moderate effects (approximately 0.27‐0.43 SD units) depending on the intracluster correlation structure.

### Study Timeline

**Figure 2.**
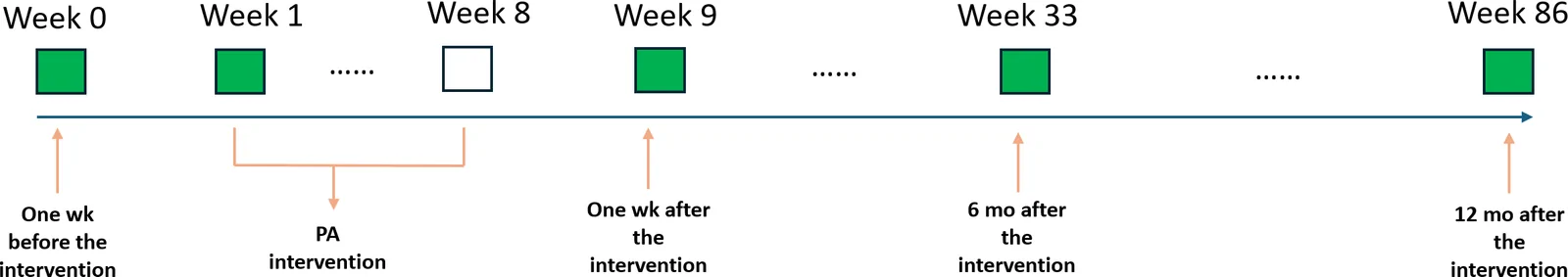
Overall study procedures. PA: physical activity.

Participants will be monitored during 5 week-long assessment periods over 21 months (Figure 2), including baseline (week 0), the first week of the program (week 1), the week immediately after the program (week 9), 6 months after the program (week 33), and 18 months after the program (week 86), to capture both short-term adoption and longer-term maintenance of PA.

Before the PA program begins (baseline; week 0), participants will visit the Center for HOPE and complete a baseline questionnaire, undergo anthropometric measurements, and receive a study smartphone and MotionSense units, along with training on the use of these study devices. During the PA program phase (weeks 1‐8), all participants will take part in the ‘Ohana Project, an 8-lesson lifestyle intervention delivered over 8 weeks to promote PA. After the intervention, participants will be followed for 18 months (weeks 8‐86) to assess their longer-term PA maintenance. Although 12 months after PA program cessation has been recommended as appropriate for studying longer-term PA maintenance [8,9], we use 18 months so that our results are comparable to those of some other studies that adopted the same follow-up period [40].

During each of the 5 observational weeks, participants will wear the MotionSense device during their waking hours (eg, 7:30 AM to 9:30 PM) for 7 consecutive days. The device samples acceleration data at 25 Hz and stores the data on the memory card. It also calculates the second-by-second Euclidean norm minus one (ENMO) values, a validated and widely adopted indicator of PA intensity [41,42], and streams it in real time to the study smartphone via the MotionPI app, an app developed by the team [24]. Each day, participants are asked to power on the smartphone when they wake up, carry both the phone and the wristbands throughout their waking hours, respond to EMAs, and power off and charge the phone before bedtime. The wristband will automatically sync with the phone when powered on. After each assessment week, participants will visit the Center for HOPE to return the equipment and complete follow-up questionnaires.

### Intervention: ‘Ohana Project

The ʻOhana Project is an 8 week–long, culturally tailored lifestyle program adapted from the Centers for Disease Control and Prevention’s Diabetes Prevention Program. It was developed to align with Pacific Islander cultural context and has been implemented previously with Pacific Islander communities in Hawaii [43,44]. This program uses evidence-based behavioral strategies to promote leisure-time PA, healthy eating, and stress management. Because the Pacific Islander population in Utah shares similar social norms, attitudes toward PA, and PA barriers with Native Hawaiians, this program is well suited for this study.

The ‘Ohana Project protocol includes an 8-lesson curriculum delivered in 8 weeks. All lessons will be taught in group sessions of approximately 15 participants at the National Tongan American Society, consistent with Pacific Islander cultural values that emphasize collective learning and social support [43,45]. The lessons will focus on PA initiation, goal setting, action planning, and strategies to cope with barriers and sustain behavior change.

### Measures

#### Overview

This study uses real-time, field-based, state-of-the-art methodologies to assess PA and its determinants in participants’ real-world environments. Data are collected using an integrated system that includes MotionSense wearable sensors, EMA, GPS tracking, and GISs. These components communicate wirelessly and synchronize data in real time via a study smartphone, enabling the simultaneous capture of objective PA behavior, subjective psychosocial states, and spatial-contextual exposures at fine temporal scales. MotionSense, EMA, and GPS data are temporally aligned and spatially linked to GIS-derived measures of the built and social environment.

This integrated approach allows for objective and continuous measurement of PA and its key correlates while minimizing participant burden, yielding higher-resolution and more ecologically valid data than traditional retrospective or aggregated assessment methods.

#### PA

PA is measured objectively using MotionSense, a pair of wrist-worn devices, each containing a triaxial accelerometer, gyroscope, and photoplethysmography sensor [23]. Participants wear the devices on both wrists, and PA is measured from the nondominant wrist. Raw accelerometer data are stored in the wristband memory. The wristband is also programmed to calculate second-by-second ENMO values [41,42] and stream them in real time to the study smartphone via the Bluetooth connection.

ENMO values are summarized using established cutoff points to derive participants’ time spent in sedentary behavior, total PA, and MVPA minutes, which will serve as the primary PA outcomes. Nonwear time is identified from the raw acceleration signal using the standard van Hees algorithm implemented in the *GGIR* package [46,47], and nonwear minutes are removed prior to summarizing PA estimates. Analyses are restricted to the protocol-defined waking hour window (7:30 AM-9:30 PM; 14 hours per day). A day is considered valid if a participant accumulates wear time equal to at least 70% of this window (≥9.8 hours per day). An assessment week is considered valid if it includes at least 4 valid days, at least one of which is a weekend day. Device failure will be detected by the quality control function of the platform, and the device will be replaced if necessary.

#### Psychosocial Acute Precipitants

Psychosocial acute precipitants are believed to be the most proximal factors to behaviors and more prone to change than other factors, therefore providing a direct path linking contextual environments to PA and informing interventions that target behavior change [26].

Psychosocial acute precipitant data are collected through the MotionPI app [24] on the smartphone. Participants use their finger to respond to EMA items on the smartphone. Each EMA contains 24 questions regarding self-efficacy, outcome expectancies, motivation for PA, social support for PA, positive affect, perceived access to PA opportunities, perceived stress, and perceived racial and ethnic discrimination.

Up to 6 EMAs will be triggered per day during the observation weeks. Specifically, each day is divided into 3 blocks (7:30 AM-noon, noon-4:30 PM, and 4:30 PM-bedtime). During each block, up to 1 EMA will be triggered via detection of PA, and 1 will be triggered at random. Thus, a minimum of 3 random EMAs will be triggered for every participant each day. EMAs triggered via PA are limited to up to 3 per day to maintain a reasonable participant burden. EMAs triggered via PA occur after detection of at least 10 minutes of MVPA [27,48,49]. The software is designed to trigger the EMA after the MVPA bouts are over so that it does not interrupt the PA participation.

#### Neighborhood Factors

Participants’ exposure to neighborhood factors will be characterized by 2 different spatial approaches: one focusing on the static neighborhood of the participants and the other focusing on participants’ movements in space. For static neighborhood, numerous spatially referenced databases will be used to describe each participant’s “living” and “working” environments. GIS layers will be clipped to participant-centered neighborhood buffers using careful aggregation schema, and a final buffer size will be determined after looking at the distribution of the residential addresses. The built and social factors of interest include PA facility proximity, public transit proximity, land use mix, street walkability, residential segregation, poverty, greenness, and social cohesion. Other neighborhood factors such as employment, housing, litter, noise, air pollution, weather, and safety also contribute to PA participation and will be examined in exploratory analyses. Key spatial datasets for measures of neighborhood function will be derived from the US census (eg, population density, poverty, educational level, and race and ethnicity at the census block or block group level); the Utah State Geographic Information Datasource, one of the most mature and extensive GIS databases in the nation (eg, appraised value of residences, 1-m level green space data, parks, hiking trails, and public transit routes and stations); city police departments (eg, crime, violence, and graffiti, geocoded at the point level); and other government agencies (eg, air quality and weather).

In terms of participants’ movement in space, their GPS points will be used to calculate key travel modes and trajectories and time spent in specific locations. Space-time variables on exposure to built environment and social factors will be calculated and synchronized with MotionSense and EMA data by aligning time stamps. A series of Euclidean buffers (eg, 0.25-mi radius) that move along the participant’s route at 1-minute increments will be evaluated. Route analysis will be restricted to the study area (ie, the Salt Lake valley) to maximize the quality and consistency of the spatial datasets. This analysis will use many of the same databases mentioned in the previous paragraph but will assess the likelihood of each participant being affected by key environmental factors within their own mobility patterns. Indexes (eg, deprivation) may be developed to reduce issues with collinearity and/or misclassification error.

#### Sociodemographic and Other Factors

Individual characteristics, including sociodemographic factors, mental health, attitudes toward PA, knowledge, emotional regulation, and PA history, will be assessed via a questionnaire and modeled as baseline predictors and potential confounders. Additionally, the study will collect BMI and body fat, functional capacity, and reasons for selecting one’s neighborhood. These measures will be assessed using anthropometric assessments, the 6-minute walk test [50], and a baseline questionnaire and will be included as potential confounders given their well-established associations with PA engagement and neighborhood selection.

### Statistical Analysis Plan

#### Aim 1 Analysis (Neighborhood Factors and PA Adoption and Maintenance)

Generalized linear mixed-effects models (GLMMs) will be used to examine associations between neighborhood built and social environment factors and PA behavior change among Pacific Islanders. Primary outcomes will include (1) PA adoption, defined as whether a participant meets the weekly threshold of 150 minutes of MVPA (binary) and (2) PA maintenance, operationalized as weekly MVPA minutes (continuous). Primary exposure will be neighborhood built and social environment factors. Models will control sociodemographic characteristics and biobehavioral predispositions. Specifically, PA adoption will be analyzed using a logistic mixed-effects model, and PA maintenance will be analyzed using a linear mixed-effects model. In both models, week of follow-up and covariates will be specified as fixed effects, and participant-specific random effects will be included to account for within-person correlation across repeated weekly observations.

In addition to the unadjusted analyses, adjusted models will be used to analyze the impact of each covariate of interest. The adjusted analysis will combine results across participant-level quintiles of the covariate of interest [51], with adjustment for other covariates that may be imbalanced across levels of the covariate of interest. Notably, the quintile strata for the prediction models will be defined using baseline measurements, whereas time-varying covariates will be predicted at a fixed time point of interest. Each prediction model will be constructed via a convex combination of machine learning algorithms. Specifically, each model will include L1-regularized regression (linear or logistic, with linear, quadratic, and interaction terms) and random forest (super learner) [52].

#### Aim 2 Analysis (Dynamic Associations and Mediation)

Dynamic prediction models (DPMs) will be used to analyze the complex, time-varying relationships between contextual and psychosocial acute precipitants and PA behavior. Compared to traditional GLMMs, DPMs can use momentary-level intensive longitudinal data to investigate complex temporal associations among multiple real-time constructs to yield a prediction model that not only signals the probability of PA ahead of time but also updates the prediction as time progresses and more recent data become available.

The DPM is a system of (theoretically) infinite models for making predictions at any time *t*0. At any time *t*0 during the observation, it predicts the likelihood of PA over a chosen future time horizon using a logistic or linear regression model, with covariates constructed from the longitudinal data observed prior to *t*0. This yields a sequence of time-indexed “static” regression models. Because dropout patterns and time-varying effects can cause model parameters to differ across time, these parameters are modeled as smooth functions of the prediction time *t*0. The DPM approach fits the entire model sequence in a single step by estimating these parameter functions while appropriately accounting for dependence across models.

Mediation analyses will be conducted within a GLMM framework consistent with the primary outcome models. Each mediation model will simultaneously include the proposed mediator (eg, psychosocial acute precipitants, such as a participant’s momentary negative affect) and the exposure of interest hypothesized to operate through that mediator (eg, contextual acute precipitants, such as a participant’s momentary green space exposure), along with additional hierarchical components that link these processes. Contextual and psychosocial acute precipitants will be aggregated over short time intervals (eg, 15 minutes) and appropriately lagged to represent participant history and preserve temporal ordering for mediation (eg, negative affect during the prior 15-minute interval). The indirect (mediated) effect will be estimated using the product-of-coefficients approach: the coefficient for the mediator in the outcome model multiplied by the coefficient for the exposure in the mediator model. For the adjusted model, prediction models will be constructed for both covariates of interest based on the other covariates with potential imbalances. Then, predictions for each will be used to form 3 groups each based on tertiles for a total of 9 groups in the above-described stratified analysis.

### Missing Data

Missing data will be addressed using multiple imputation [53]. We will generate 10 imputed datasets using predictive mean matching with 50 iterations per dataset. Multiple imputation can ensure that analyses are valid if the data are missing at random. To examine the potential impact of missingness not at random, we will perform sensitivity analyses under several hypothesized configurations of missingness not at random by extending the techniques in the work by Siddique et al [54].

### Ethical Considerations

This study protocol has been reviewed and approved by the University of Utah Institutional Review Board (00164610). Written informed consent will be obtained prior to enrollment. All participants will receive financial compensation for their time.

### Dissemination Plan

Information on methodological and measurement strategies, descriptive results, and statistical analyses will be disseminated via working papers, scholarly presentations, and publications. The results will also be shared with the community and public health partners via brief reports, presentations, and other communication materials.

## Results

This study was funded by the National Cancer Institute of the National Institutes of Health in August 2023. Data collection started on May 23, 2025, and is expected to be finished in March 2028. As of August 10, 2026, the project has recruited all 150 participants. Data collection and analysis are ongoing, and results are expected to be published in May 2028.

## Discussion

### Anticipated Findings

Although empirical evidence specific to Pacific Islanders remains limited, findings from studies of other racial and ethnic minority populations provide important guidance [55]. We expect that neighborhood poverty, land use mix, and proximity to public transit will be among the strongest neighborhood-level predictors of PA adoption and maintenance among Pacific Islanders. At the momentary level, we expect that self-efficacy, negative affect, and access to PA resources will be key determinants of PA engagement. We further expect that self-efficacy and negative affect will mediate the effects of contextual factors on PA and that psychosocial mechanisms underlying PA will differ between the adoption and maintenance stages, consistent with prior empirical and theoretical research.

### Comparison to Prior Work

This paper describes the protocol of a novel longitudinal mHealth study examining PA behavior change among Pacific Islanders using both static and momentary measures. To the authors’ knowledge, it is among the first studies to focus on PA adoption and maintenance for this population group. By integrating a comprehensive set of neighborhood built and social environment measures with contextual and acute psychosocial precipitants, the study will deepen our understanding of both individual- and neighborhood-level barriers to and facilitators of PA participation and generate actionable evidence to inform interventions aimed at reducing PA-related health burdens for Pacific Islanders.

The study is also among the first to leverage real-time, real-world data on PA alongside momentary contextual exposures and psychosocial states to investigate PA behavior change. Using an intensive and state-of-the-art mHealth system to capture contextual and acute psychosocial precipitants at fine temporal scales, we will examine how psychosocial factors interact with momentary contexts to shape PA behaviors. In addition, advanced DPMs will be applied to characterize temporal dynamics between predictors and PA. By leveraging high-frequency mobile assessment data, these models can capture moment-to-moment relationships, enabling both insight into risk-outcome pathways and more accurate risk prediction than static approaches.

### Strengths and Limitations

The primary strength of this study is its comprehensive multilevel design, which integrates objective and subjective measures, real-time and real-world assessment, short- and long-term follow-up, and both individual- and neighborhood-level determinants to examine PA behavior change among Pacific Islanders, an approach that has been rarely implemented in minority health research. In addition, engagement of the target community through community advisory board members participating across multiple stages, including study development, pilot-testing, recruitment, and implementation, enhances the cultural relevance, feasibility, and sustainability of the research. Finally, the multidisciplinary research team with expertise in epidemiology, engineering, psychology, sociology, geography, and Pacific Islander health supports rigorous analyses and culturally informed interpretation of the findings.

The study has several limitations. First, the generalizability of our findings may be limited to the Pacific Islanders included in this study, specifically Tongan Americans. Pacific Islanders are a diverse population, and cultural norms may differ across groups, such as Tongans, Samoans, and Chamorros. We selected a relatively homogeneous subgroup to reduce internal heterogeneity, but this choice involves a trade-off with external generalizability. Future studies should examine whether these findings extend to other Pacific Islander subpopulations. Second, our previous focus group study with Tongan Americans found that 92% (33/36) of participants expressed interest in using exercise to improve their health, suggesting that our sample may be more motivated than the broader population. To address potential biases due to motivation, a “motivation for PA” item was added to our questionnaires and EMA assessments, and we will closely monitor the proportion and dropout rate for both motivated and unmotivated participants and assess and adjust for their influences on PA outcomes across time.

## Supplementary material

10.2196/108686 Peer Review Report 1 Peer review report from the National Cancer Institute (NCI), part of the US National Institutes of Health (NIH), under grant R37CA276365.

## Abbreviations

DPM: dynamic prediction model
EMA: ecological momentary assessment
ENMO: Euclidean norm minus one
GIS: geographic information system
GLMM: generalized linear mixed-effects model
mHealth: mobile health
MVPA: moderate to vigorous physical activity
PA: physical activity
PiPA: Pacific Islander Physical Activity
