# Supplementary material for: Using Momentary Measures to Understand Physical Activity Adoption and Maintenance: Protocol for a Longitudinal Study
Source: JMIR Res Protoc. 2026 Sep 25;15:e108686. doi: 10.2196/108686 (PMC13614216; doi:10.2196/108686)
Supplement: Supplementary file 1 [file resprot-v15-e108686-s001.pdf]

SUMMARY STATEMENT
(Privileged Communication)

PROGRAM CONTACT:
David Berrigan
(240) 276-6752
berrigad@mail.nih.gov

Release Date: **02/27/2023**
Revised Date:

Application Number: **1 R01 CA276365-01A1**
Formerly: **1R01CA276365-01A1**

Principal Investigator

WAN, NENG

Applicant Organization: UNIVERSITY OF UTAH

Review Group: **CIHB**
Community Influences on Health Behavior Study Section

Meeting Date: **02/14/2023**
Council: **MAY 2023**
Requested Start: **07/01/2023**
RFA/PA: **PA20-185**
PCC: **6BHP**
Dual IC(s): **MD**

Project Title: **Using Momentary Measures to Understand Physical Activity Adoption and Maintenance among Pacific Islanders in the United States**
SRG Action: **Impact Score:20 Percentile:5**
Next Steps: **Visit https://grants.nih.gov/grants/next_steps.htm**
Human Subjects: **30-Human subjects involved - Certified, no SRG concerns**
Animal Subjects: **10-No live vertebrate animals involved for competing appl.**
Gender: **1A-Both genders, scientifically acceptable**
Minority: **2A-Only minorities, scientifically acceptable**
Age: **3A-No children included, scientifically acceptable**

Project Year	Direct Costs Requested	Estimated Total Cost
1	498,644	757,325
2	499,213	758,189
3	495,315	752,269
4	499,807	759,091
5	497,348	755,356
TOTAL	2,490,327	3,782,230

ADMINISTRATIVE BUDGET NOTE: The budget shown is the requested budget and has not been adjusted to reflect any recommendations made by reviewers. If an award is planned, the costs will be calculated by Institute grants management staff based on the recommendations outlined below in the COMMITTEE BUDGET RECOMMENDATIONS section.
EARLY STAGE INVESTIGATOR
NEW INVESTIGATOR

1R01CA276365-01A1 Wan, Neng

EARLY STAGE INVESTIGATOR
NEW INVESTIGATOR

RESUME AND SUMMARY OF DISCUSSION: In this highly-responsive resubmitted R01 application, the investigators propose to examine the influence of contextual, environmental and psychosocial factors on physical activity adoption and maintenance among Pacific Islander adults in Utah. During discussion, committee members agreed that the proposed project has great potential to advance the understanding of contextual and psychological influences on physical activity, and to have a public health impact among an underserved population at risk for physical inactivity and related outcomes. The study is supported by rigorous prior research and the combined use of sensors, ecological momentary assessment and GPS is innovative. Led by a promising new/early-stage investigator with experience in the field, the multidisciplinary investigative team has complementary expertise and a history of collaboration which position them to successfully conduct the proposed research. The research environments at the University of Utah and Ohio State University are also outstanding for the work. Committee members were enthusiastic about the project's clear conceptual model; comprehensive assessment of physical activity and built environment; engagement with the Tongan community; and plans for dissemination. Committee members noted minor weaknesses around potential implications for future intervention, a lack of detail regarding consent processes and generalizability to other Pacific Islander populations but these did not diminish their enthusiasm for the application. Following discussion, committee members agreed that the proposed project will make a major contribution to the field of physical activity among at-risk populations.

DESCRIPTION (provided by applicant): Pacific Islanders represent one of the fastest growing racial/ethnic groups in the United States. Pacific Islanders suffer from disparities in a variety of health problems such as obesity, cardiovascular diseases, diabetes, and cancer at several sites. Although physical activity is an effective way to reduce the risks of these health problems, Pacific Islander adults are generally less physically activity than non-Hispanic whites. The search for effective policies and interventions to promote PA among Pacific Islanders is severely hampered by the paucity of research on the mechanisms underlying PA behavioral change in this population. This longitudinal study will examine the influence of contextual and environmental factors and acute momentary precipitants on PA adoption and maintenance among 150 sedentary Pacific Islander adults. This study is guided by an overarching conceptual framework derived from models of the social/environmental determinants of health, social cognitive theories of behavioral change, and prior empirical findings. Participants will be assessed using real-time, field-based, state of the art methodologies consisting of MotionSense, ecological momentary assessment, and GPS. MotionSense track behavioral and physiologic data in real-time and can objectively detect PA behaviors and negative affect/stress of participants. GPS permits real-time mapping of an individual's space-time trajectories and relevant environmental exposures/characteristics (e.g., proximity to PA facilities; neighborhood safety) using geographic information system data. Principal outcomes of interest are PA adoption and PA maintenance. This research would be the first to systematically study PA behaviors of Pacific Islander adults and the first to combine objective and dynamic indices of PA, negative affect/stress, and key environmental influences in PA behavior studies. The comprehensive, multi-method approach is a major advance for the field as it eliminates problems related to an exclusive reliance on self-reports and static residential locations for key outcomes and predictors. In addition, this is one of the first studies to include the framework of dynamic prediction models, a novel statistical approach, to fully examine the huge amount of data yielded by real time assessment approaches. The results will provide critical evidence to guide policies and interventions targeted at reducing PA-related health disparities experienced by Pacific Islanders and potentially other racial/ethnic minorities.

PUBLIC HEALTH RELEVANCE: This project will examine the influences of neighborhood built and social environments, acute individual and contextual precipitants, as well as demographics and social history, biobehavioral and psychosocial predispositions on physical activity (PA) adoption and maintenance among sedentary Pacific Islander adults in Utah. It will use emerging mobile health technologies to measure PA and its key correlates in real time in the real world. The study will fill key gaps in the literature on important correlates and mechanisms underlying PA behavioral change for this fast-growing, high health risk, yet severely understudied population group, and will provide critical information on how to improve their PA level and reduce obesity and obesity-related health problems. The methodology and findings derived from this study will also provide a new perspective in understanding PA behavior of other racial/ethnic groups with high obesity and low PA levels.

CRITIQUE 1

Significance: 2
Investigator(s): 2
Innovation: 3
Approach: 2
Environment: 1

Overall Impact: This application seeks to examine the influences of neighborhood factors and momentary contextual and psychosocial factors on adoption and maintenance of physical activity among sedentary Pacific Islander adults in Utah. The team is composed of a well-rounded group of investigators, the research environment is supportive, and the National Tongan American Society is engaged to inform translation efforts. The topic is important and significant in an underserved population, even though the findings are specific to a distinct group (Tongan American adults in Utah) and not necessarily generalizable to all Pacific Islanders adults. There are methodological innovations and use of rigorous methods (e.g., careful attention to objective activity measures in space, use of mHealth tools). The previous weaknesses have mostly been addressed (examples include addressing the influence of motivation, addressing subject burden, with others discussed in the Resubmission section below), although not all of them can be fully addressed or resolved. Nonetheless, mitigation approaches have been implemented to address the inherent study weaknesses such that they are viewed as minor weaknesses. Successful completion of the aims is expected to make a contribution of high importance to the field, with minor weaknesses.

1. Significance:

Strengths

- Examining health behaviors in an underserved population that suffers disproportionate poor health outcomes from physical inactivity.
- Focus on contextual social and built environment factors (macro-level) on activity participation.

Weaknesses

- There is still a lack of generalizability to Pacific Islander adults in the U.S. more broadly because the study will focus on Tongan Americans in Utah. The investigators acknowledge this limitation and given the lack of information on physical activity and contextual factors among this population, addressing the aims should still provide important information to add to the field. This is viewed as a minor weakness and is not a major score driving weakness. (minor)

- The time frame is still short (a little less than 2-years) and therefore the findings will be limited; adoption and especially maintenance of physical activity needs to be examined over the long term. This reviewer appreciates the trade-offs that must be made in any study and the points that the investigators made in their resubmission. Once again, the general lack of research on this topic in this population suggests that even short-term studies may provide some important information, so this is considered a minor (not a major score driving) weakness. (minor)

2. Investigator(s):

Strengths

- Application from NI/ESI with experience in the field.
- Interdisciplinary team.
- Collaboration of National Tongan American Society with letter of support.

Weaknesses

- None noted.

3. Innovation:

Strengths

- Employing a suite of tools for comprehensive activity assessment.
- Examining dynamic relationships between real-time experiences (EMA) and activity levels.
- Solid measures of environmental factors.

Weaknesses

- While there are methodological innovations, the aims and hypotheses are not that innovative. However, these research questions are not well studied in the Tongan population, so this is viewed as a minor weakness. (minor)

4. Approach:

Strengths

- Engagement of the Tongan community in translation.
- Strong recruitment and retention plans, with cultural considerations.
- Rigorous, objective measures of physical activity and the built environment.

Weaknesses

- There remains an issue of the observational measures imbedded within an intervention because the sample will be a group who are motivated to improve their PA levels. However, the investigators note some strategies to mitigate this potential influence in the resubmission (e.g., survey item on motivation for PA and use in statistical adjustment, monitor proportion of drop-outs by motivation status). (minor)

5. Environment:

Strengths

- More than adequate.

Weaknesses

- None noted.

Protections for Human Subjects:

Acceptable Risks and/or Adequate Protections

- No concerns noted.

Data and Safety Monitoring Plan (Applicable for Clinical Trials Only):

Not Applicable (No Clinical Trials)

Inclusion Plans:

- Sex/Gender: Distribution justified scientifically
- Race/Ethnicity: Distribution justified scientifically
- Inclusion/Exclusion Based on Age: Distribution justified scientifically
- No concerns noted.

Vertebrate Animals:

Not Applicable (No Vertebrate Animals)

Biohazards:

Not Applicable (No Biohazards)

Resubmission:

- The investigators did a good job of responding to the previous reviewer concerns, including addressing the following: study design and its strengths and weaknesses, length of follow-up, generalizability, influence of motivation on outcomes, influence of planned/scheduled activity, conceptual model, measurement, recruitment, and participant burden.

Resource Sharing Plans:

Acceptable

Budget and Period of Support:

Recommend as Requested

CRITIQUE 2

Significance: 2
Investigator(s): 2
Innovation: 2

Approach: 3
Environment: 2

Overall Impact: In this cross-sectional study, investigators will examine influences of neighborhood factors on physical activity adoption and maintenance and examine their dynamic relationships. The proposal is both significant and innovative - from the rigor of prior research, clearly explained conceptual model, and novel application to the population under study and methodological approaches. There are weaknesses that are all judged to be minor. The application was responsive to the prior reviews. The team is well-equipped to conduct this research in has a strong community partnership. Together, strengths outweigh weaknesses in this proposal deemed to have the potential for high impact.

1. Significance:

Strengths

- Justified rationale for examination of neighborhood influences of PcIs specifically
- Clear conceptual model outlining relationships, including the larger scope and specific cope to be examined in this study
- High rigor of prior research surrounding psycho-social factors, and their specific repetitive measurement needs, and contextual/neighborhood factors as they relate to physical activity

Weaknesses

- Rationale for increasing physical activity does not need to rely upon arguments related to obesity and is advantageous in its own right

2. Investigator(s):

Strengths

- ESI investigator with sufficient and training and track record leading confidence to the successful execution of this project
- Evidence of successful collaboration among team members
- CoIs with extensive experience with grants of similar size and scope

Weaknesses

- None noted.

3. Innovation:

Strengths

- Well delineated aspects of methodological innovation and population specific innovation.

Weaknesses

- None noted.

4. Approach:

Strengths

- Partnership with National Tongan American Society provides confidence in attaining recruitment goals. Will also host the ‘Ohana group sessions will hopefully add to adherence to protocol
- Community Advisory Board to provide feedback about on-going study implementation
- Preliminary data and pilot testing plan lends confidence to measurement feasibility
- Justified study timeline and data collection period
- Sex as a biological variable will be explored in analysis phase

Weaknesses

- Explanation of how the sensor detects stress is not written in generally accessible language
- Figure 1 (Conceptual model) is not referred back to in the proposal (measurement, analytic plans)
- Participant burden could be further lessened if research team travel to their place of residence to collect data instead of having them come in or by zoom
- It’s possible that more thorough explanation/comprehension checks/pilot testing of the consent language may be needed to ensure comprehension of the somewhat complex data procedures that participants will be consenting to in order to sufficiently understand the risks involved

5. Environment:

Strengths

- Environment is well resourced and supportive of the proposed research plan
- Study team directs relevant centers and labs (such as the Utah Geo Health Lab and the Center for Health Outcomes and Population Health Equity)

Weaknesses

- None noted.

Protections for Human Subjects:

Acceptable Risks and/or Adequate Protections

- Sufficient protections are in place

Data and Safety Monitoring Plan (Applicable for Clinical Trials Only):

Not Applicable (No Clinical Trials)

Inclusion Plans:

- Sex/Gender: Distribution justified scientifically
- Race/Ethnicity: Distribution justified scientifically
- Inclusion/Exclusion Based on Age: Distribution justified scientifically
- All inclusion plans are sufficient

Vertebrate Animals:

Not Applicable (No Vertebrate Animals)

Biohazards:

Not Applicable (No Biohazards)

Resubmission:

- Sufficient response to reviewers' comments in this resubmission

Resource Sharing Plans:

Acceptable

Budget and Period of Support:

Recommend as Requested

CRITIQUE 3

Significance: 2
Investigator(s): 1
Innovation: 1
Approach: 1
Environment: 1

Overall Impact: This resubmission proposes to examine the influences of neighborhood, momentary contextual, and psychosocial factors on physical activity adoption and maintenance among Pacific Islanders in Utah. Specifically, investigators propose to: (1) implement a physical activity intervention for 150 Pacific Islander Americans, a group at high risk for low physical activity, obesity, and associated health problems; and (2) during the intervention, and follow up period, roll out a battery of measures for capturing the role of contextual, psychological and psychobiological variables in shaping participant uptake and maintenance of physical activity, including a GIS approach, via cell phones, that will allow for the assessment of environmental features of geographic locations on a moment to moment basis, along with a physiological assessment of stress-related biomarkers. If successfully implemented, the overall impact could be high. This research will provide important information on contextual and psychological influences on physical activity research in an underserved and under researched high-risk community, Pacific Islanders. Strengths of the proposal include methodological innovations and use of rigorous methods (e.g., careful attention to objective activity measures in space, use of MotionSense mHealth tool), an investigative team with a wealth of experience in the related necessary areas, a supportive environment to conduct this work, and interaction with the National Tongan American Society for translation efforts. The changes made to the proposal, along with additional clarifications and details provided confirm the potential for this application to provide potentially relevant findings regarding physical activity levels in an at-risk population that could form the basis of longer-term studies of activity adoption and maintenance in this and potentially other at-risk populations.

1. Significance:

Strengths

- Examining health behaviors in Pacific Islander Americans, a vulnerable but understudied population that suffers disproportionate poor health outcomes from physical inactivity.
- Focus on contextual social and built environment factors (macro-level) on activity participation (but with a tie-in to individual level factors).
- Little previous research in this group or other groups that have used a combination of environmental data captured by GIS technology, along with psychological variables, to capture moment to moment influences on behavior.
- High rigor of prior research about what is known about contextual and psychosocial influences on physical activity in general, and among Pacific Islanders in particular; need for more delineated/specific information about physical activity habits in real-world; differentiation between PA adoption and maintenance.

Weaknesses

- The proposed project, while having potential to address a gap in science, does not lead very clearly to new intervention approaches. The investigators suggest that it could result in policies to increase green space. It is already well known that green space is related to health.

2. Investigator(s):

Strengths

- Application from NI/ESI with experience in the field.
- Very strong interdisciplinary team that includes experts in geography, GIS, mobile health technology, exercise physiology, etc.
- PI Wan has funded work from the ACS and an institutional pilot grant in these areas.
- Collaboration history between CoIs/PI.
- Community Advisory Board and partnership with National Tongan American Society (NTAS).

Weaknesses

- None noted.

3. Innovation:

Strengths

- Employing a suite of tools for comprehensive activity assessment.
- Examining dynamic relationships between real-time experiences (EMA) and activity levels.
- Although GIS technologies are not novel, this application will draw on a much more precise approach to identify individuals' location in space from moment to moment, which can then be linked to neighborhood variables as the individual moves through space and time.
- Combining local environmental variables with measures of stress, using non self-report methods, seems novel, and the mediational model proposed to reflect relationships among these variables is also novel.
- Population focus and use of mHealth technologies.

Weaknesses

- None noted.

4. Approach:

Strengths

- Choice of a vulnerable and understudied population is a strength.
- Engagement of the Tongan community in translation.
- Application convincing that recruitment and retention will be feasible.
- Strong recruitment and retention plans, with cultural considerations.
- Rigorous, objective measures of physical activity and the built environment.
- Use of an existing physical activity intervention to assess physical activity uptake and maintenance is a strength.
- Proposed conceptual model, outlining a mediational relationship between environmental variables, physical activity access, and psychological stress, and physical activity uptake
- Preliminary study regarding the mHealth system (with even more sensors than proposed in this study) demonstrated lack of major non-compliance issues and willingness to use mHealth approaches among the target population.
- Measurement of physical activity allows for real-time EMA and for evaluation of different physical activity intensity levels.
- Pilot test planned (n=20).

Weaknesses

- None noted.

5. Environment:

Strengths

- This is a very strong environment. The study will be carried out by researchers affiliated with several research groups at the University of Utah, which is a leading center of research on GIS influences on health, exercise science, and other relevant research areas.
- University of Utah (prime) and Ohio State University provide outstanding resources to support this project, in particular the mHealth and computing aspects.
- Strong Letter of support from O. Fahina Tavake-Pasi from the National Tongan American Society.

Weaknesses

- None noted

Study Timeline:

Strengths

- Adequate timeline

Weaknesses

- None noted

Protections for Human Subjects:

Acceptable Risks and/or Adequate Protections

Data and Safety Monitoring Plan (Applicable for Clinical Trials Only):

Not Applicable (No Clinical Trials)

Inclusion Plans:

- Sex/Gender: Distribution justified scientifically
- Race/Ethnicity: Distribution justified scientifically
- Inclusion/Exclusion Based on Age: Distribution justified scientifically

Vertebrate Animals:

Not Applicable (No Vertebrate Animals)

Biohazards:

Not Applicable (No Biohazards)

Resubmission:

- Incorporated relevant papers to better justify time frame.
- To address potential biases due to motivation, investigators will now include an item of 'Motivation for PA' in questionnaires and EMA assessments, and closely monitor the proportion of motivated participants and assess/adjust for its influence on PA outcomes across time.
- Added items of planned/scheduled PA in questionnaires and EMAs, and will adjust for its influence in data analysis.
- Added a figure to show how MotionSense looks like.
- Provided more information and clarification on 1) the conceptual model diagram (to better delineate the pathways, the mediation analysis, and the focus of the study), 2) measurement of negative/stress, 3) recruitment of participants and CAB members, 4) participant burden, mitigation procedures, and compliance, 5) overlap of expertise between Haaland (i.e., statistics) and Wen (i.e., health sociology and neighborhood effect analysis), 6) the importance of PA on reducing the risks of obesity and other chronical diseases, and 7) how this study differentiates from other studies that focuses on one or two of the factors.

Resource Sharing Plans:

Acceptable

Budget and Period of Support:

Recommend as Requested

THE FOLLOWING SECTIONS WERE PREPARED BY THE SCIENTIFIC REVIEW OFFICER TO SUMMARIZE THE OUTCOME OF DISCUSSIONS OF THE REVIEW COMMITTEE, OR REVIEWERS' WRITTEN CRITIQUES, ON THE FOLLOWING ISSUES:

PROTECTION OF HUMAN SUBJECTS: ACCEPTABLE

INCLUSION OF WOMEN PLAN: ACCEPTABLE

INCLUSION OF MINORITIES PLAN: ACCEPTABLE

INCLUSION ACROSS THE LIFESPAN: ACCEPTABLE

COMMITTEE BUDGET RECOMMENDATIONS: The budget was recommended as requested.

Footnotes for 1 R01 CA276365-01A1; PI Name: Wan, Neng

NIH has modified its policy regarding the receipt of resubmissions (amended applications).See Guide Notice NOT-OD-18-197 at https://grants.nih.gov/grants/guide/notice-files/NOT-OD-18-197.html. The impact/priority score is calculated after discussion of an application by averaging the overall scores (1-9) given by all voting reviewers on the committee and multiplying by 10. The criterion scores are submitted prior to the meeting by the individual reviewers assigned to an application, and are not discussed specifically at the review meeting or calculated into the overall impact score. Some applications also receive a percentile ranking. For details on the review process, see http://grants.nih.gov/grants/peer_review_process.htm#scoring.

MEETING ROSTER

Community Influences on Health Behavior Study Section
Healthcare Delivery and Methodologies Integrated Review Group
CENTER FOR SCIENTIFIC REVIEW
CIHB
02/14/2023 - 02/15/2023

Notice of NIH Policy to All Applicants: Meeting rosters are provided for information purposes only. Applicant investigators and institutional officials must not communicate directly with study section members about an application before or after the review. Failure to observe this policy will create a serious breach of integrity in the peer review process, and may lead to actions outlined in NOT-OD-22-044 at https://grants.nih.gov/grants/guide/notice-files/NOT-OD-22-044.html, including removal of the application from immediate review.

CHAIRPERSON(S)

RAMSAWAMY, MEGHA, PHD
PROFESSOR
DEPARTMENT OF POPULATION HEALTH
SCHOOL OF MEDICINE
UNIVERSITY OF KANSAS
KANSAS CITY, KS 66160

MEMBERS

BAQUERO, BARBARA I, PHD, MPH *
ASSOCIATE PROFESSOR
DEPARTMENT OF HEALTH SYSTEMS AND POPULATION HEALTH
UNIVERSITY OF WASHINGTON
SEATTLE, WA 98105

BONAR, ERIN E, PHD
ASSOCIATE PROFESSOR
DEPARTMENT OF PSYCHIATRY
UNIVERSITY OF MICHIGAN
ANN ARBOR, MI 48109

COCKBURN, MYLES G, PHD *
PROFESSOR
DEPARTMENT OF POPULATION
AND PUBLIC HEALTH SCIENCES
KECK SCHOOL OF MEDICINE
UNIVERSITY OF SOUTHERN CALIFORNIA
LOS ANGELES, CA 90089

DUBOWITZ, TAMARA, SCD *
SENIOR POLICY RESEARCHER
RAND CORPORATION
FACULTY PARDEE RAND GRADUATE SCHOOL
PITTSBURGH, PA 15213

DUNCAN, GLEN E, PHD
PROFESSOR AND CHAIR
DEPARTMENT OF NUTRITION AND EXERCISE PHYSIOLOGY
ELSON S. FLOYD COLLEGE OF MEDICINE
WASHINGTON STATE UNIVERSITY
SPOKANE, WA 99202

GANZ, OLLIE, DRPH *
INSTRUCTOR
DEPARTMENT OF HEALTH BEHAVIOR, SOCIETY AND POLICY
RUTGERS SCHOOL OF PUBLIC HEALTH
NEW BRUNSWICK, NJ 08901

GOHLKE, JULIA M, PHD
ASSOCIATE PROFESSOR
DEPARTMENT OF POPULATION HEALTH SCIENCES
VIRGINIA POLYTECHNIC INSTITUTE
AND STATE UNIVERSITY
BLACKSBURG, VA 24061

GOINGS, TRENETTE C, PHD
PROFESSOR
SCHOOL OF SOCIAL WORK
THE UNIVERSITY OF NORTH CAROLINA AT CHAPEL HILL
CHAPEL HILL, NC 27599

GOODKIND, JESSICA R, PHD
PROFESSOR
DEPARTMENT OF SOCIOLOGY
UNIVERSITY OF NEW MEXICO
ALBUQUERQUE, NM 87131

GUEST, MARC ARRON, MPH, PHD, MSN *
ASSISTANT PROFESSOR
CENTER FOR INNOVATION IN HEALTHY
AND RESILIENT AGING
ARIZONA STATE UNIVERSITY
PHOENIX, AZ 85004

HAN, INKYU, PHD *
ASSOCIATE PROFESSOR
DEPARTMENT OF EPIDEMIOLOGY AND BIOSTATISTICS
COLLEGE OF PUBLIC HEALTH
TEMPLE UNIVERSITY
PHILADELPHIA, PA 19122

HARRELL, MELISSA B, PHD *
PROFESSOR
DEPARTMENT OF EPIDEMIOLOGY HUMAN GENETICS AND ENVIRONMENTAL SCIENCE
UTHEALTH HOUSTON, SCHOOL OF PUBLIC HEALTH
AUSTIN, TX 78701

HIRATSUKA, VANESSA Y, PHD
ASSISTANT PROFESSOR AND CO-DIRECTOR
CENTER FOR HUMAN DEVELOPMENT
UNIVERSITY OF ALASKA ANCHORAGE
ANCHORAGE, AK 99508

JACQUEZ, FARRAH M, PHD *
PROFESSOR
DEPARTMENT OF CLINICAL PSYCHOLOGY
UNIVERSITY OF CINCINNATI
CINCINNATI, OH 45221

KELLY, BRIAN C, PHD
PROFESSOR
DEPARTMENT OF SOCIOLOGY
PURDUE UNIVERSITY
WEST LAFAYETTE, IN 47907

LEBRON, ALANA MW, PHD *
ASSISTANT PROFESSOR
DEPARTMENT OF HEALTH, SOCIETY AND BEHAVIOR
DEPARTMENT OF CHICANO/LATINO STUDIES
SCHOOL OF SOCIAL SCIENCES
UNIVERSITY OF CALIFORNIA, IRVINE
IRVINE, CA 92697

MARSHALL, BRANDON DL, PHD
ASSOCIATE PROFESSOR
DEPARTMENT OF EPIDEMIOLOGY
SCHOOL OF PUBLIC HEALTH
BROWN UNIVERSITY
PROVIDENCE, RI 02912

MARTIN, MOLLY A, MD
PROFESSOR
DEPARTMENT OF PEDIATRICS
UNIVERSITY OF ILLINOIS CHICAGO
CHICAGO, IL 60612

MASSEY, PHILIP M, PHD
ASSOCIATE PROFESSOR
DEPARTMENT OF COMMUNITY HEALTH SCIENCES
FIELDING SCHOOL OF PUBLIC HEALTH
UNIVERSITY OF CALIFORNIA, LOS ANGELES
LOS ANGELES, CA 90095

MINNIS, ALEXANDRA M, PHD *
SENIOR RESEARCH EPIDEMIOLOGIST
RESEARCH TRIANGLE INSTITUTE INTERNATIONAL
BERKELEY, CA 94704

MOJTABAI, RAMIN, PHD
PROFESSOR
DEPARTMENT OF MENTAL HEALTH
BLOOMBERG SCHOOL OF PUBLIC HEALTH AND
DEPARTMENT OF PSYCHIATRY AND BEHAVIORAL SCIENCES
JOHNS HOPKINS UNIVERSITY
BALTIMORE, MD 21205

PAHL, KERSTIN, PHD
RESEARCH ASSOCIATE PROFESSOR
DEPARTMENT OF PSYCHIATRY
SCHOOL OF MEDICINE
NEW YORK UNIVERSITY
NEW YORK, NY 10016

QUINTILIANI, LISA M, PHD
ASSOCIATE PROFESSOR
DEPARTMENT OF MEDICINE
SECTION OF GENERAL INTERNAL MEDICINE
BOSTON UNIVERSITY SCHOOL OF MEDICINE
BOSTON, MA 02118

SCARINCI, ISABEL C, PHD
PROFESSOR
DEPARTMENT OF OBSTETRICS AND GYNECOLOGY
UNIVERSITY OF ALABAMA AT BIRMINGHAM
BIRMINGHAM, AL 35294

THRASHER, JAMES, PHD
PROFESSOR
DEPARTMENT OF HEALTH PROMOTION,
EDUCATION AND BEHAVIOR
ARNOLD SCHOOL OF PUBLIC HEALTH
UNIVERSITY OF SOUTH CAROLINA
COLUMBIA, SC 29208

WARD, TONY J, PHD
PROFESSOR AND CHAIR
SCHOOL OF PUBLIC
AND COMMUNITY HEALTH SCIENCES
UNIVERSITY OF MONTANA
MISSOULA, MT 59812

WHITE, JUSTIN S, MPH, PHD *
ASSOCIATE PROFESSOR
DEPARTMENT OF EPIDEMIOLOGY AND BIOSTATISTICS
SCHOOL OF MEDICINE
UNIVERSITY OF CALIFORNIA, SAN FRANCISCO
SAN FRANCISCO, CA 94158

WILLIAMS, EDITH M, PHD
ASSOCIATE PROFESSOR
DEPARTMENT OF PUBLIC HEALTH SCIENCES
AND ALLERGY-IMMUNOLOGY-RHEUMATOLOGY
UNIVERSITY OF ROCHESTER MEDICAL CENTER
ROCHESTER, NY 14642

SCIENTIFIC REVIEW OFFICER

MCREE, ANNIE LAURIE, DRPH
SCIENTIFIC REVIEW OFFICER
CENTER FOR SCIENTIFIC REVIEW
NATIONAL INSTITUTES OF HEALTH
BETHESDA, MD 20892

EXTRAMURAL SUPPORT ASSISTANT

WATTS, MELISSA D
EXTRAMURAL SUPPORT ASSISTANT
CENTER FOR SCIENTIFIC REVIEW
NATIONAL INSTITUTE FOR HEALTH
BETHESDA, MD 20892

* Temporary Member. For grant applications, temporary members may participate in the entire meeting or may review only selected applications as needed.

Consultants are required to absent themselves from the room during the review of any application if their presence would constitute or appear to constitute a conflict of interest.
